# Single-Stranded DNA Binding Proteins and Their Identification Using Machine Learning-Based Approaches

**DOI:** 10.3390/biom12091187

**Published:** 2022-08-26

**Authors:** Jun-Tao Guo, Fareeha Malik

**Affiliations:** Department of Bioinformatics and Genomics, University of North Carolina at Charlotte, Charlotte, NC 28223, USA

**Keywords:** single-stranded DNA, ssDNA, single-stranded DNA binding protein, SSB, binding specificity

## Abstract

Single-stranded DNA (ssDNA) binding proteins (SSBs) are critical in maintaining genome stability by protecting the transient existence of ssDNA from damage during essential biological processes, such as DNA replication and gene transcription. The single-stranded region of telomeres also requires protection by ssDNA binding proteins from being attacked in case it is wrongly recognized as an anomaly. In addition to their critical roles in genome stability and integrity, it has been demonstrated that ssDNA and SSB–ssDNA interactions play critical roles in transcriptional regulation in all three domains of life and viruses. In this review, we present our current knowledge of the structure and function of SSBs and the structural features for SSB binding specificity. We then discuss the machine learning-based approaches that have been developed for the prediction of SSBs from double-stranded DNA (dsDNA) binding proteins (DSBs).

## 1. Introduction

Since Watson and Crick’s Nobel Prize winning discovery in 1953, the canonical representation of genomic DNA conformation has been the double-stranded DNA (dsDNA) helical structure [1]. However, there are many instances that single-stranded DNA (ssDNA) exists either transiently or consistently. For example, dsDNA unwinds to form ssDNA during essential biological processes such as DNA replication, transcription, recombination, and repair [2,3,4,5,6]. Unlike the double-helical structure of dsDNA that is stabilized by base pairing and base stacking, ssDNA is more flexible and less stable, making it vulnerable to chemical or enzymatic attacks [3,7]. The 3′ single-stranded DNA overhang, a key component of telomere structure at the end of eukaryotic chromosomes, is susceptible to “unauthorized” processing or misrecognition as DNA damage [3,8]. The lack of protection of these ssDNA regions can pose serious problems to genomic stability and integrity and may cause diseases [8,9].

The critical importance of maintaining genome stability and the need of protecting vulnerable ssDNA from damages require a special family of proteins, called ssDNA binding proteins (SSBs), which are ubiquitous in all living organisms [2,4]. The first SSB was discovered and characterized in the bacteriophage T4 in 1970 [10]. Shortly after that, the *E*. *coli* SSB protein was identified [11,12]. *E*. *coli* SSB, which functions as a homotetramer, is the most widely studied prokaryotic SSB. Replication protein A (RPA), originally identified as a key component for simian virus 40 (SV40) replication, represents the first eukaryotic SSB that was found to be directly involved in DNA metabolism [13,14,15,16]. Human RPA is a heterotrimeric complex composed of three subunits, RPA70, RPA32, and RPA14, and is involved in a variety of DNA repair pathways including mismatch repair, double-stranded break repair, and recombination repair [17]. Two other human SSBs, termed hSSB1 and hSSB2, were identified in 2008 [18]. It has been demonstrated that hSSB1 plays important roles in genome stability as well as in cell cycle regulation and transcription [7,18].

Another group of SSBs that are of paramount importance in maintaining genome stability is telomere end-binding protein (TEBP), a sequence-specific ssDNA binding protein. At the very end of eukaryotic chromosomes of the 3′ termini, there exist single-stranded DNA overhangs. These overhangs vary in length in different species, ranging from several to hundreds of bases [5,9]. To safeguard these vulnerable 3′ overhangs from inappropriate processing, TEBP binds and acts as a cap to sequester the ssDNA in a sequence-specific manner [5,8]. Failure of such protection can lead to destabilization of the genome and early onset of cellular senescence [8,19,20].

## 2. SSB Structure, Binding Specificity and Function

### 2.1. Structural Folds of ssDNA Binding Domains

There are four main structural folds in ssDNA binding domains: oligonucleotide/oligosaccharide-binding (OB) folds, K homology (KH) domains, RNA recognition motifs (RRMs), and whirly domains [2]. The OB fold is a well-known ssDNA binding domain found in many SSBs, including *E. coli* SSB, bacteriophage T4 SSB, human RPA, human mtSSB, human SSB1 and SSB2, and the telomere-end protection family, such as TEBP from *Oxytricha nova*, Cdc13 from *Saccharomyces cerevisiae*, and Pot1 from humans.

The OB fold consists of a five-stranded antiparallel β-sheet that forms a characteristic β-barrel core with various lengths of loops connecting the strands [2,21]. The narrow ssDNA binding cleft on the surface of the β-barrel is close to strand 2 and strand 3 [2]. ssDNA binds its interacting surface with its bases facing the protein. The number and organization of the OB domains that participate in ssDNA interaction vary quite differently [22]. RPA is a heterotrimer with six OB folds, two for subunit interaction and four for ssDNA binding. *E. coli* SSB is a homotetramer with each unit having one OB domain. The SSB from *Sulfolobus solfataricus* (ssoSSB) has a single OB domain and binds ssDNA with a footprint of five bases and a defined binding polarity [22].

**Figure 1:**
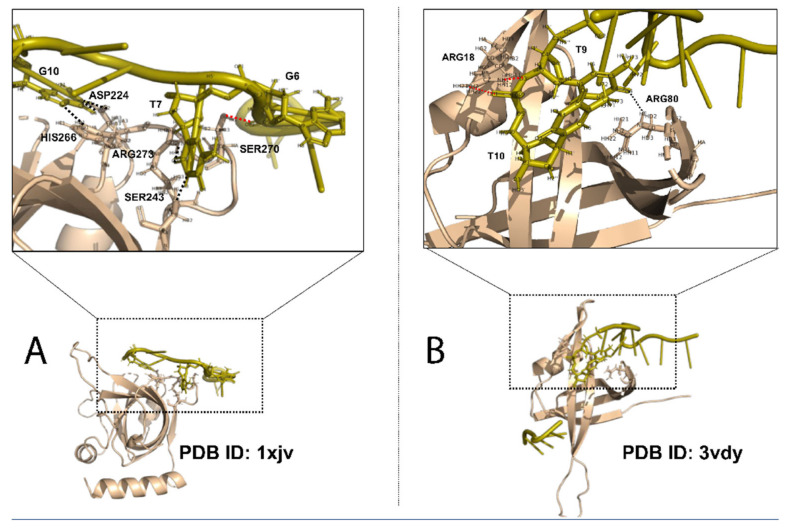
OB fold of SSBs and binding specificity. (**A**) Specific OB fold domain of human POT1 bound to telomeric single-stranded DNA (TTAGGGTTAG), PDB ID: 1xjv, chain: A, domain: 151–299. (**B**) Non-specific OB fold of *Bacillus subtilis* SsbB, PDB ID: 3vdy, chain: A. Black dashed lines, sidechain-base hydrogen bonds; red dashed lines, non-sidechain-base hydrogen bonds.

The diversity and versatility of the OB fold can be understood from two different angles. First, OB fold proteins have different ssDNA binding specificity (Figure 1). Even though binding specificity is a relative term and is difficult to define with a hard cutoff, proteins can be grouped based on the differences of binding affinity among a large number of sequences [23]. In both protein-dsDNA interaction and protein-ssDNA interaction, hydrogen bonds (HBs) between DNA bases and protein sidechain atoms are considered the major contributor to binding specificity [23,24,25,26,27,28,29,30,31]. Some OB fold proteins bind ssDNA with very high sequence specificity. The prime example of sequence-specific OB fold is TEBP proteins that recognize specific sequences at the 3′ ssDNA overhang at the end of chromosomes [2]. The complex structure between human POT1 OB fold domain and telomeric ssDNA (TTAGGGTTAG) reveals more sidechain-base HBs than non-sidechain-base HBs (Figure 1A) [32]. Some other OB fold proteins are sequence independent or non-specific, meaning they can bind different ssDNA sequences. The non-specific ssDNA binding OB folds include the highly conserved eukaryotic RPA and bacteria SSBs [33]. Figure 1B shows the non-specific binding between *Bacillus subtilis* SsbB OB fold and ssDNA, which has a fewer percentage of sidechain-base HBs [34]. The binding specificity also depends on the sequence/structure context. For example, the C-terminal of the ORF6 of *Enterobacter Phage* Enc 34, is important for specific binding to ssDNA. The removal of the C-terminal from Enc 34 makes it less specific as it can bind both ssDNA and dsDNA [35]. The second aspect of the OB fold diversity lies in its lack of sequence conservation. While the sequence diversity of the OB fold provides a variety of interaction surface for recognition of different ssDNA sequences, especially for the sequence-specific OB-fold SSBs, it presents challenges in identification of new SSBs based on sequence information alone.

The KH domains are characterized by three α-helices that are closely packed with a three-stranded β- sheet. ssDNA binds to KH domains with conserved polarity and with bases facing the protein. Examples of ssDNA binding KH domains include hnRNP k, FBP, poly (C)-binding protein (PCBP) 1 and 2 [2]. All of these KH domains bind ssDNA at sites upstream of promoter regions and affect transcription. Although the RRM domains are abundant in annotated human genomes, there are only five proteins with RRM domains that have known structures complexed with ssDNA, including human UP1, RBM-45, FBP-interacting repressor (FIR), and SUP-12 and MEC-8 from *Caenorhabditis elegans* [36,37,38,39]. Compared to other three ssDNA binding domains, whirly domains are relatively large, comprising about 180 amino acids. They have 2 four-stranded β-sheets arranged in almost parallel along with some helical fragments. The whirly domains are mostly found in mitochondria and chloroplasts in plants and perform diverse functions in transcriptional activation, splicing, and DNA repair.

### 2.2. Structural Features in SSB Binding Specificity

As described previously, SSBs can bind ssDNA specifically or non-specifically. While there are quite a number of studies on structural features in protein-dsDNA binding specificity [23,24,26,40,41,42,43,44], the specificity investigation of protein-ssDNA interaction is underexplored due to the limited availability of protein-ssDNA complex structures. We recently performed a comparative study of protein–ssDNA interactions in terms of binding specificity [27]. A non-redundant dataset of SSB–ssDNA complexes with high structural quality was generated and classified into two groups: specific and non-specific, based on their binding specificity, which was manually annotated by searching the primary references of these SSB–ssDNA complexes and their homologs in Protein Data Bank (PDB) [45,46] as well as relevant information in UniProt [47]. We then compared the key structural features in protein-ssDNA interaction, including binding propensities and secondary structure types of ssDNA base-interacting residues, hydrogen bonds, π–π interactions between residue side chains and DNA bases, interaction interfaces, and protein conformational changes upon ssDNA binding [27].

**Figure 2:**
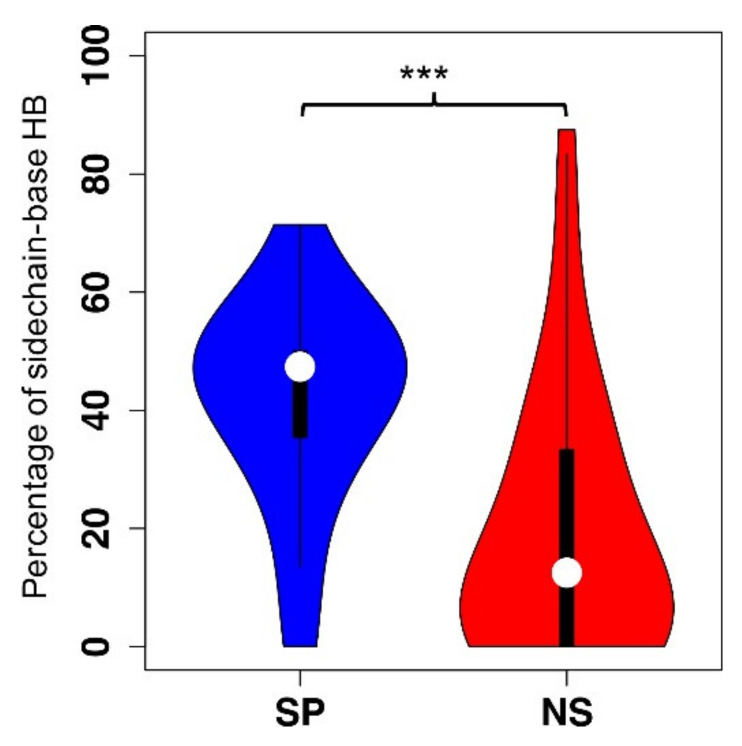
Comparison of the distribution of the percentage of the sidechain-base hydrogen bonds in each protein chain–ssDNA complex between the specific (SP) and the non-specific (NS) protein–ssDNA complexes. Statistical analysis of the comparison between the NS and SP groups was done with Wilcoxon rank sum test. *** = *p*-value ≤ 0.001.

Hydrogen bonds and amino acid binding propensities were found to be the key discriminating features between specific and non-specific ssDNA binding proteins [27] (Figure 2 for comparison of sidechain-base hydrogen bonds). As for amino acid binding propensities, while aromatic and positively charged amino acids, phenylalanine, tryptophan, tyrosine, histidine, lysine, and arginine are enriched in both specific and non-specific protein-ssDNA complexes, three amino acids (histidine, tyrosine, and arginine) are more enriched in the specific group than those in the non-specific group. The positively charged lysine and arginine are capable of forming both hydrogen bonds with DNA bases and ionic interactions with the negatively charged DNA backbone atoms. Aliphatic amino acids alanine, isoleucine, leucine, and valine show low propensity in both binding specificity groups. Interestingly, the negatively charged aspartate is also enriched in the specific protein-ssDNA interactions as we demonstrated in specific protein-dsDNA interactions [23,27]. π–π interactions may primarily contribute to binding affinity as there are no apparent difference of π–π interactions between the two binding specificity groups. No significant differences were found between specific and non-specific groups with respect of conformational changes upon ssDNA binding, suggesting that the flexibility of SSBs plays a lesser role than that of double-stranded DNA-binding proteins in conferring binding specificity. The features that are different between specific and non-specific ssDNA binding proteins can be applied to machine learning algorithms for prediction of binding specificity if the protein–ssDNA complex structures are available.

Wang et al. carried out a comparative analysis of structural features that can be used to differentiate SSBs and DSBs [48]. The analysis was based on a dataset of 238 DSBs and 97 SSBs in complex with dsDNA and ssDNA respectively. The features include surface shapes that are grouped into three categories: peak, flat, and valley, and the surrounding environment, such as amino acid composition and electrostatic charge of the interface. They demonstrated that the distributions of the above features are significantly different between DSBs and SSBs, which can be used for structure-based classification between DSBs and SSBs [48].

### 2.3. Role of ssDNA and SSBs in Transcription Regulation

In addition to their critical roles in genome stability and integrity, it has been demonstrated that ssDNA and SSBs play important roles in transcriptional regulation in all three domains of life and viruses [49,50,51,52,53,54,55,56,57,58,59,60]. In the traditional model of transcriptional regulation, transcription factors recognize and bind to their cognate DNA binding sites in double helical form to either activate or repress gene expression [24]. However, it has been shown that the presence of single-stranded DNA regions in a number of transcriptionally active promoters is much more than previously thought, which warranted an investigation of their role in the regulation of gene transcription [49]. Attempts to unravel the mechanism of ssDNA in transcriptional regulation not only revealed that they are important for optimal transcription but also led to the identification of a number of SSBs. For example, c-myc transcription is regulated by the binding of a sequence-specific SSB, FBP (FUSE-binding protein), to FUSE (far upstream element) [50,61]. Heterogeneous nuclear ribonucleoprotein (hnRNP) K binds to single-stranded DNA with a CT element upstream of the c-myc promoter and activates transcription [62]. In mice, sequence-specific SSBs were reported to be involved in the transcriptional regulation of μ-opioid receptor gene and timp-1 gene (54,55). A number of unique ssDNA binding proteins that participate in transcriptional regulation have also been identified in plants [56,57,58,59]. Desveaux et al. found a novel ssDNA binding protein that regulates the expression of the PR-10a gene in potato [56]. The protein, called PBF-2 for PR-10a binding factor 2, binds to a 30-bp promoter sequence with high affinity in a sequence-specific manner. PBF-2 consists of four p24 subunits that belong to a novel family of ubiquitous plant-specific whirly family [58]. In Arabidopsis, the Whirly1 (Why1) protein not only binds to transcriptional response elements, it also binds to telomeric DNA and modulates telomere length homeostasis [63].

### 2.4. SSBs and Diseases

Aberrant expressions and changes in SSBs can result in DNA damage and lead to cancer [17,64]. Conditional deletion of mouse homolog of hSSB1 in mice showed increased susceptibility to several types of tumors [65]. It has been shown that defective regulation and expression of APOBEC3A (apolipoprotein B messenger RNA-editing enzyme, catalytic polypeptide-like), a single-stranded DNA deoxycytidine deaminase, is involved in the development of cancer [66,67]. BRCA2, a breast cancer tumor suppressor, promotes direct interactions between RAD51 recombinase and ssDNA. Loss-of-function mutations in BRCA2 increase susceptibility to breast and ovarian cancers owing to genome instability [68,69,70]. Moreover, small molecules that inhibit binding activity between ssDNA and RPA can prevent cell cycle progression and enhance chemosensitivity in cancer cells, suggesting a therapeutic value for targeting SSBs [17,71].

## 3. Machine Learning-Based Methods for SSB Prediction

Despite the critical roles of ssDNA binding proteins in essential biological processes, investigation of protein–ssDNA interactions clearly lags behind other types of protein-nucleic acids interactions, such as protein–dsDNA interactions, due to the limited number of protein–ssDNA complex structures. Another key limiting factor in studying protein–ssDNA interactions is that new SSBs have been identified at a very slow pace. As indicated in a recent call of an open invitation to the Understudied Proteins Initiative [72,73], in the life science field, research efforts focus on a group of well-studied proteins, while the biological function of the majority of proteins are understudied or remain unexplored due to a variety of practical reasons. These proteins with unknown functions are termed “the dark matter of the sequence universe”, and it is practically impossible to experimentally characterize each of them from the dark [74,75,76,77]. Therefore, it is of particular importance to develop efficient computational methods for predicting new SSBs from the uncharacterized proteins. Not only can it expand the landscape of SSBs, it can also narrow down the cases to a reasonable number for follow-up experimental validations and potentially increase the number of ssDNA–SSB complex structures for structural studies.

**Figure 3:**
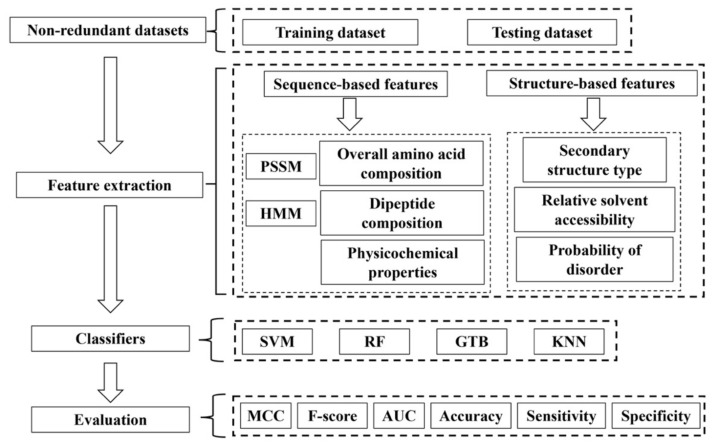
The flowchart for machine learning-based prediction of SSBs.

Machine learning methods have been widely used in a number of research topics in structural bioinformatics. The application of machine learning methods to bioinformatics problems culminated in the success of the development of AlphaFold for protein structure prediction, a scientific breakthrough that was built upon years of previous efforts [78,79,80,81]. Various machine learning methods have also been developed for prediction of nucleic acid-binding or DNA binding proteins from protein sequences [82,83,84,85,86,87,88,89,90,91,92,93,94]. Compared to the large number of publications on prediction of DNA binding proteins, the investigation on ssDNA binding protein prediction is limited so far. To our knowledge, currently there are only four published studies on SSB prediction using machine learning-based approaches [95,96,97,98]. These methods typically consist of four major steps as shown in Figure 3: (1) dataset generation for training and testing; (2) features for learning and prediction; (3) classification models; and (4) performance evaluation. We discuss below each of these four steps in machine learning-based methods for SSB prediction.

### 3.1. Datasets

Prediction performance of machine learning-based approaches, especially when applying the learning models to real-world biological problems, relies on carefully generated, robust data sets for training and testing [94]. So far, the prediction of SSBs has been generally carried out for differentiation between SSBs and dsDNA binding proteins (DSBs). Essentially all the methods use the same training and independent test sets from the work by Wang et al. [95,96,97,98]. The training set (Uniprot1065) consists of 873 DSBs and 183 SSBs, which were culled from UniProtKB/SwissProt with a sequence identity cutoff at 0.7. The independent non-redundant dataset of SSBs and DSBs was derived from PDB with 125 DSBs and 41 SSBs, which share less than 30% sequence identity [95]. These protein structures were either solved by X-ray crystallography with a resolution of less than 3 Å or by NMR.

It is understandable that a higher sequence identity cutoff was adopted for the training dataset construction due to limited availability of annotated SSBs. However, it may cause an overfitting problem in machine learning models as evidenced by the unbalanced performance between the training set and the independent dataset, resulting in a big drop of MCC (Matthew’s correlation coefficient) from the training set to the independent set, regardless of the type of models or features used for the prediction [95,96,97,98]. Another potential issue that may contribute to the unbalanced performance is the phenomenon of protein moonlighting since some DNA binding proteins can bind both ssDNA and dsDNA. For example, in the independent dataset, *Arabidopsis* cryptochrome 3 (2VTBE) and the tumor repressor p53 (2B3GB) are annotated to bind both ssDNA and dsDNA [99,100,101,102]. In addition, some proteins may be able to bind both RNA and DNA or they are RNA binding proteins, but due to technical challenges in solving RNA structures, the structures were solved with ssDNA instead of ssRNA.

Two other datasets were mentioned in SSB prediction studies. Sharma et al. compiled an additional independent test set with 64 SSBs and 53 DSBs, as shown in their GitHub site after filtering out cases with more than 70% sequence identity with the above mentioned training set and independent set by Wang et al. [96]. Tests were performed with three sequence identity cutoffs at 0.9, 0.7, and 0.3 for this new independent test set [96]. We performed a CD-HIT analysis of this new set with a 0.3 sequence identity cutoff and found that there are only 18 SSBs and 14 DSBs, while 13 out of the 47 entries were annotated as double-stranded RNA binding proteins and 2 were considered as ssDNA and dsDNA binding proteins in UniProt [70]. Therefore, the low prediction performance may be a result of this dataset with mixed annotations. Tan et al. mentioned a dataset with 1271 SSBs and 2252 DSBs in their study, which includes the training set from Wang.et al. [98]. However, it is not clear how the proteins in the list were selected and the number of false positive SSBs as the proteins of this dataset are not published.

### 3.2. Features

Both sequence features and sequence-derived structural features have been applied for SSB and DSB prediction (Table 1 and Figure 3). The widely-used sequence features in protein structure and function prediction, such as PSSM (position specific scoring matrix) and HMM (hidden Markov model) profiles, which consider evolution information and are generated from multiple sequence alignments, have been explored [95,97,98]. PSSM is typically generated with PSI-BLAST through searching a protein database to detect increasingly divergent members of a protein in consecutive iterations [103,104].

Both the original PSSM or PSSM derivatives have been used for prediction of SSBs and DSBs. For example, a feature descriptor called consensus sequence-based K-segmentation PSSM (CSKS-PSSM) was developed in SDBP-Pred [97]. To generate the consensus sequence, a position in a protein sequence is replaced with a residue with the maximum substitution probability at this position. For K-segmentation PSSM, the PSSM is split into K-segments of equal sizes. Ali et al. implemented two- and three-segmented PSSM in their model [97]. Sharma et al. used HMM profiles produced from HHblits to capture evolutionary information [96]. More specifically, they applied the normalized profile-monogram and normalized profile-bigram feature extraction methods, which have been demonstrated to be useful in protein fold prediction and intrinsically disordered region prediction [96].

Other sequence features that have been explored for SSB and DSB prediction include overall amino acid composition (OAAC), dipeptide composition, and physicochemical properties [95,98]. OAAC is a 20-dimensional descriptor with a frequency value for each of the 20 standard amino acids for a given protein sequence. It has been indicated that using the square root of the frequencies can result in a better performance than the raw frequencies [95,105]. Dipeptide compositions represent the frequencies of two consecutive residues, or two residues separated by one or two residues in a protein sequence. Wang et al. and Tan et al. used all three types of dipeptide compositions that are separated by zero, one, or two residues [95,98]. As for the physicochemical properties, 28 descriptors have been selected from over 500 physicochemical and biochemical properties in the AAindex database for classification between SSBs and DSBs [95,98,106].

In addition to the sequence-based descriptors, some sequence-derived structural features have been used for classification between SSBs and DSBs, such as the predicted secondary structure types, relative solvent accessibility, and the probability being disordered [98]. The prediction accuracy of secondary structure types improved greatly with deep learning approaches. DeepCNF, a deep learning-based secondary structure prediction method, has achieved a three-state accuracy of 84% [107]. With the availability of AlphaFold, we expect to see that structural features will play more roles in future prediction of DNA binding proteins.

### 3.3. Classification Models

Several traditional machine learning methods, such as random forests (RF) [108] and support vector machines (SVM) [109] have been used in SSB predictions [95,96,97] (Table 1 and Figure 3). RF and SVM are two very popular traditional machine learning methods. They have been extensively used for protein structure and function prediction with good performance before the era of deep learning. Random forest is an ensemble learning method with many decision trees for both classification and regression problems. Compared to the simple decision tree method, it has a low risk of overfitting. Random forest methods have been applied for predicting protein–protein interactions, protein structure prediction, and function prediction, such as prediction of DNA binding proteins [88,89,110,111,112]. Support vector machines use hyperplane separation and kernel function optimization to classify a given dataset [113]. SVM methods have been used in improving the prediction of protein secondary structure types, protein fold recognition, and functional prediction [83,85,114,115,116]. There are different types of kernel functions that can be used for SVM classification, including linear, polynomial, radial basis function (RBF), and sigmoid. In SSB prediction, while some studies only used one kernel function, such as using the default parameters in Wang et al. or RBF in Sharma et al., Ali et al. tried three kernel functions: linear, polynomial, and RBF [95,96,97]. For the RF methods, the number of trees was set at 3000 in both RF models [95,96]). Tan et al. used gradient tree boosting (GTB) for classification and the parameters were determined by a 10-fold cross-validation based on the training set with a grid search approach [97]. The performances of these classifiers are summarized in the next section.

### 3.4. Performance Evaluation

The performances of the predictive models, a combination of classifiers and weighted features, were evaluated with the widely used measurements for classification problems (Figure 3). These measurements include accuracy, sensitivity, specificity, MCC, AUC (area under the ROC curve), and F-score (or F1-score). These values were calculated on both the training set and the independent test set. To estimate the performance of machine learning models, usually a 10-fold cross validation method was applied to the training dataset before assessing the models on the independent test set [95,96,97,98]. Among the four aforementioned classification models for SSB prediction, there is a mixed performance even with the same set of feature descriptors, datasets, and classifier models. With HMM profiles, it seems that SVM performs slightly better than the RF and KNN methods [96]. However, with a combination of OAAC, dipeptide composition, physicochemical properties, and PSSM, Wang et al. found that RF is a better predictor than SVM [95].

## 4. Challenges and Perspective

In structure-based studies of SSBs, one major challenge is the limited number of known protein–ssDNA complexes in PDB. Machine learning-based approaches can play an important role in identifying novel SSBs and accordingly increasing the number of structures of SSBs and SSB–ssDNA complexes in follow-up studies. For sequence-based SSB prediction, currently all the published studies predict SSBs from DSBs with an assumption that we already know that a protein being predicted is a DNA-binding protein. However, to be practically useful, the more challenging problem is to predict if any given protein is a SSB or not. To improve prediction performance, as discussed above, well-designed, carefully annotated, and robust DNA binding protein datasets for training and testing are necessary. In addition, a carefully considered balance between the number of cases and redundancy is critical for developing a better prediction model. Novel sequence-based features including sequence-based structural features need to be developed for better classification or prediction. Lastly, deep learning methods have been successful recently in structural bioinformatics, such as in prediction of protein secondary structure types, solvent accessibility and disorder regions, and protein structure prediction [78,79,81,107]. However, the deep learning models typically require a large number of training protein sequences to avoid overfitting. Therefore, novel strategies need to be developed for creative use of deep learning technology for SSB prediction.

## Figures and Tables

**Table 1:** Summary of machine learning-based SSB prediction methods.

References	Predictor (If Any)	Features	Classifiers
Wang et al. [95]	NA	-OAAC -Dipeptide composition -Physicochemical properties -PSSM	SVM RF
Ali et al. [97]	SDBP-Pred	-PSSM -CS-PSSM -CSS-PSSM2 -CSS-PSSM3	SVM
Tan et al. [98]	PredPSD	-OAAC -Dipeptide composition -Physicochemical properties -PSSM -Structural features from NetSurfP and DisEMBL	GTB
Sharma et al. [96]	NA	-HMM	SVM
		-normalized profile-monogram	RF
		-normalized profile-bigram	KNN
